# Discussion on the optimization of personalized medication using information systems based on pharmacogenomics: an example using colorectal cancer

**DOI:** 10.3389/fphar.2024.1516469

**Published:** 2025-01-14

**Authors:** Mengying Yuan, Yuankun Zheng, Fei Wang, Niuniu Bai, Haoling Zhang, Yuan Bian, Hao Liu, Xia He

**Affiliations:** ^1^ Department of Pharmacy, Sichuan Academy of Medical Sciences and Sichuan Provincial People’s Hospital, University of Electronic Science and Technology of China, Chengdu, China; ^2^ Shanxi Bethune Hospital, Shanxi Academy of Medical Sciences, Third Hospital of Shanxi Medical University, Tongji Shanxi Hospital, Taiyuan, China; ^3^ Department of Pharmacy, Yuncheng Central Hospital, Yuncheng, China; ^4^ Personalized Drug Therapy Key Laboratory of Sichuan Province, School of Medicine, University of Electronic Science and Technology of China, Chengdu, China; ^5^ Department of Oncology, Sichuan Academy of Medical Sciences and Sichuan Provincial People’s Hospital, University of Electronic Science and Technology of China, Chengdu, China

**Keywords:** pharmacogenomics, colorectal cancer, personalized medication, information system, pharmaceutical service

## Abstract

Pharmacogenomics (PGx) is a powerful tool for clinical optimization of drug efficacy and safety. However, due to many factors affecting drugs in the real world, PGx still accounts for a small proportion of actual clinical application scenarios. Therefore, based on the information software, pharmacists use their professional advantages to integrate PGx into all aspects of pharmaceutical care, which is conducive to promoting the development of personalized medicine. In this paper, the establishment of an information software platform is summarized for the optimization of a personalized medication program based on PGx. Taking colorectal cancers (CRC) as an example, this paper also discusses the role of PGx in different working modes and participation in drug management of CRC patients by pharmacists with the help of information systems. Finally, we summarized the recommendations of different PGx guidelines to provide reference for the follow-up personalized pharmaceutical care.

## 1 Introduction

Pharmacogenomics (PGx) denotes the relationship between human genetic polymorphisms and the efficacy as well as safety of drugs (Corpas et al., 2024). It guides rational medication by investigating the impacts of genomic or genetic variations on the absorption, distribution, metabolism, excretion, and adverse drug reactions (ADRs) of drugs within the human body (Sadee et al., 2023). The focus of PGx research primarily encompasses genes associated with drug-metabolizing enzymes, drug transporters, and drug receptors. By elucidating the sequences and expression variations of these three gene categories, it is possible to assess the efficacy, excretion profiles, and adverse effects of pharmaceuticals (Caudle et al., 2014). Large-scale studies have demonstrated that patients who undergo genotype-guided adjustments of medication doses have a significantly lower incidence of ADRs compared with those who do not refer to the genetic results, and large-scale pharmacogenomic testing is conducive to enhancing medication safety (Swen et al., 2023). Nevertheless, PGx testing remains relatively infrequent in clinical practice. Currently, the focus of PGx testing is primarily on analyzing single drug-gene interactions. Drug-drug-gene interactions caused by multi-drug therapy (Peñas-Lledó and Llerena, 2023), along with other factors such as clinical, environmental, and individual ones, can also influence the efficacy and safety of drugs. Hence, it is indispensable to integrate these multiple influencing factors, including PGx, through an information system to optimize drug treatment and provide multi-faceted reference information for personalized and precise medication administration for patients.

## 2 Information platforms assist to optimize personalized medication regimens

At present, the research procedures of PGx are roughly based on the reported genetic polymorphisms and mutations in public databases, in combination with the genomic or genetic determination data of the observed subjects, to screen for possible variant genes. With the increasingly extensive application of deep sequencing technology, more variations will be identified in PGx, and the indexing and annotation of these variants have also enriched public database resources such as the Clinical Pharmacogenetics Implementation Consortium (CPIC), the Pharmacogenomics Knowledge Base (PharmGKB), the Dutch Pharmacogenetics Working Group (DPWG), the Pharmacogene Variation (PharmVar), the Clinical Genome Resource (ClinGen), and ClinVar (Caudle et al., 2014; Landrum et al., 2014; Rehm et al., 2015; Barbarino et al., 2018; Hulshof et al., 2022b; Ramsey et al., 2022). The resources of these public databases have enabled PGx to play an increasingly significant role in personalized medical treatment. Personalized medicine is a therapeutic strategy based on an individual’s genetic and epigenetic information (Singh, 2020). Through genetic testing, physicians can assess an individual’s genomic polymorphisms prior to prescribing medication, thereby optimizing drug selection and formulating reasonable doses and treatment courses. Currently, most of the clinical research works and applications on PGx are based on existing databases, which are mostly composed of populations from Europe and the United States. It is well known that in PGx research, there are significant differences in drug effects among different races. This disparity may be caused by pharmacokinetic and/or pharmacodynamic factors and has been verified through genes related to drug metabolism. Although numerous environmental factors influence the differences in drug responses among races, genetic factors play a crucial role. Currently, worldwide research in pharmacogenomics has made it possible for genetic testing to assess an individual’s risk of contracting diseases. Simultaneously, with the escalating application of whole-exome and whole-genome high-throughput sequencing, the volume of data is increasing significantly, which will undoubtedly result in the discovery of more common and rare genetic variations that affect disease phenotypes. Consequently, the associations between drugs and genetics will become even more complex. The research methods targeting a small number of genes/loci in the past are unable to support a much larger research system. Thus, it is necessary to employ informatic softwares to manage larger volumes of genetic data and patient information, facilitating PGx in providing precise medication recommendations for clinical practice.

**FIGURE 1:**
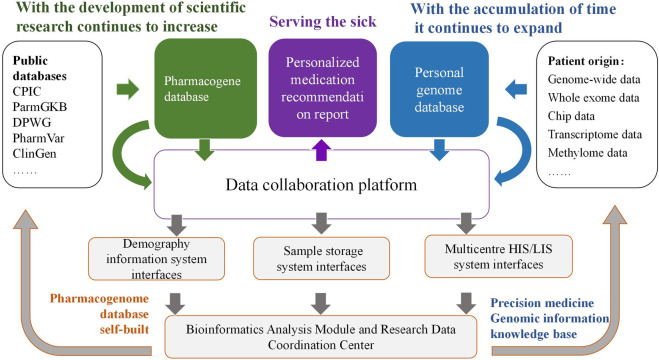
Construction of collaboration platform for PGx data analysis and application of personalized medication.

The roles of DNA diagnostics and electronic medical records in modern medical practice are increasingly prominent. The remarkable advancements in PGx have resulted in a considerable increase in the data productivity of many laboratories (Lagoumintzis et al., 2010). Through the integration of this information, we are enabled to conduct an in-depth exploration of the influence of genomic variations on human health. For instance, based on the patient data derived from systems such as the Hospital Information System (HIS) and Laboratory Information System (LIS) in multiple hospitals in China, we can establish a PGx data storage and collaborative management platform. Firstly, through the extraction and storage of medication information for various patient groups in multiple centers, the genomic sequencing of patient populations, the bioinformatics analysis of their genomic variation sites and drug genomes, as well as the new annotation and revision of existing drug genomic databases, data can be continuously accumulated to realize a PGx database specific to different population. Secondly, this database will be stored on a collaborative platform for bioinformatics analysis of PGx data, which is constructed based on C programming language, Practical extraction and report language, Python, R project, and bioinformatics analysis processes. Subsequently, genome-wide association studies (GWAS) can be conducted based on this platform to identify loci of PGx information specific to different population. Eventually, pharmacists can apply the data of this platform before patients use drugs. Utilizing the PGx data of the population, they can assist the clinic in choosing suitable drugs, formulating appropriate doses and treatment cycles. Through continuous data accumulation, analysis, and subsequent follow-ups, doses can be optimized, therapeutic effects can be enhanced, and ADRs can be reduced. Personalized medication suggestions based on the PGx database of the Chinese population can be provided for clinical practice. The specific workflow is shown in Figure 1. This system incorporates patients’ genetic information in the information mode and is applicable to medication management in multiple disease patterns. After integration with electronic medical records, an information-sharing platform for personalized clinical medication recommendations can be established, achieving the personalized medication goal of 1 + 1 > 2 and being used in the clinical decision support system. In addition, protecting the safety of genetic information is an important prerequisite for the implementation of PGx research, and the ethical safety issues involved in the practice management of PGx should also be paid attention to. On the one hand, in clinical practice, it is necessary to protect patients’ right to participate, to be informed and to make self-decisions in PGx testing; on the other hand, in information management, it is necessary to ensure that the huge database containing genetic information has reasonable authority settings and information security management.

## 3 PGx-guided pharmacy working model-an example using colorectal cancer

CRC is the third most prevalent malignant tumor worldwide, and its mortality rate is only surpassed by that of lung cancer (Sung et al., 2021), imposing a significant disease burden. Among patients diagnosed with CRC, 20% have metastatic CRC (mCRC), which carries a poor prognosis with a 5-year survival rate lower than 20% (Biller and Schrag, 2021). Traditional chemotherapeutic drugs serve as the cornerstone of drug therapy. Targeted agents and immunotherapy are emerging as one of the main therapeutic tools to improve CRC prognosis However, the inter-individual heterogeneity remains an issue in the management of CRC patients, making PGx-based individualized dosing imperative. There are numerous obstacles in applying PGx to practice. Giri et al. contend that factors such as complex and non-standard vocabularies, limited drug guidelines based on PGx, rapidly evolving evidence, inadequate awareness of the availability and practicality of test results, and inadequate accessibility for pharmacists or physicians to obtain the latest research advances for clinical services have restricted the application and development of PGx in the clinical setting (Giri et al., 2018). In practical work, we consider that the overall sample size of clinical research on PGx is relatively small, and limited real-world influencing factors (such as the pathophysiological state of patients, lifestyle and dietary habits, and drug interactions) are incorporated. Certain research results have relatively low guiding efficacy when applied to real clinical scenarios. Furthermore, pharmacogenomics accounts for only 20%–30% of the causes of inter-individual differences in drug response, with the remaining 70%–80% attributable to environmental factors (Lauschke et al., 2024). The analysis of these multiple factors affecting medications with the assistance of informational software can facilitate the clinical individualization of medication regimens.

**FIGURE 2:**
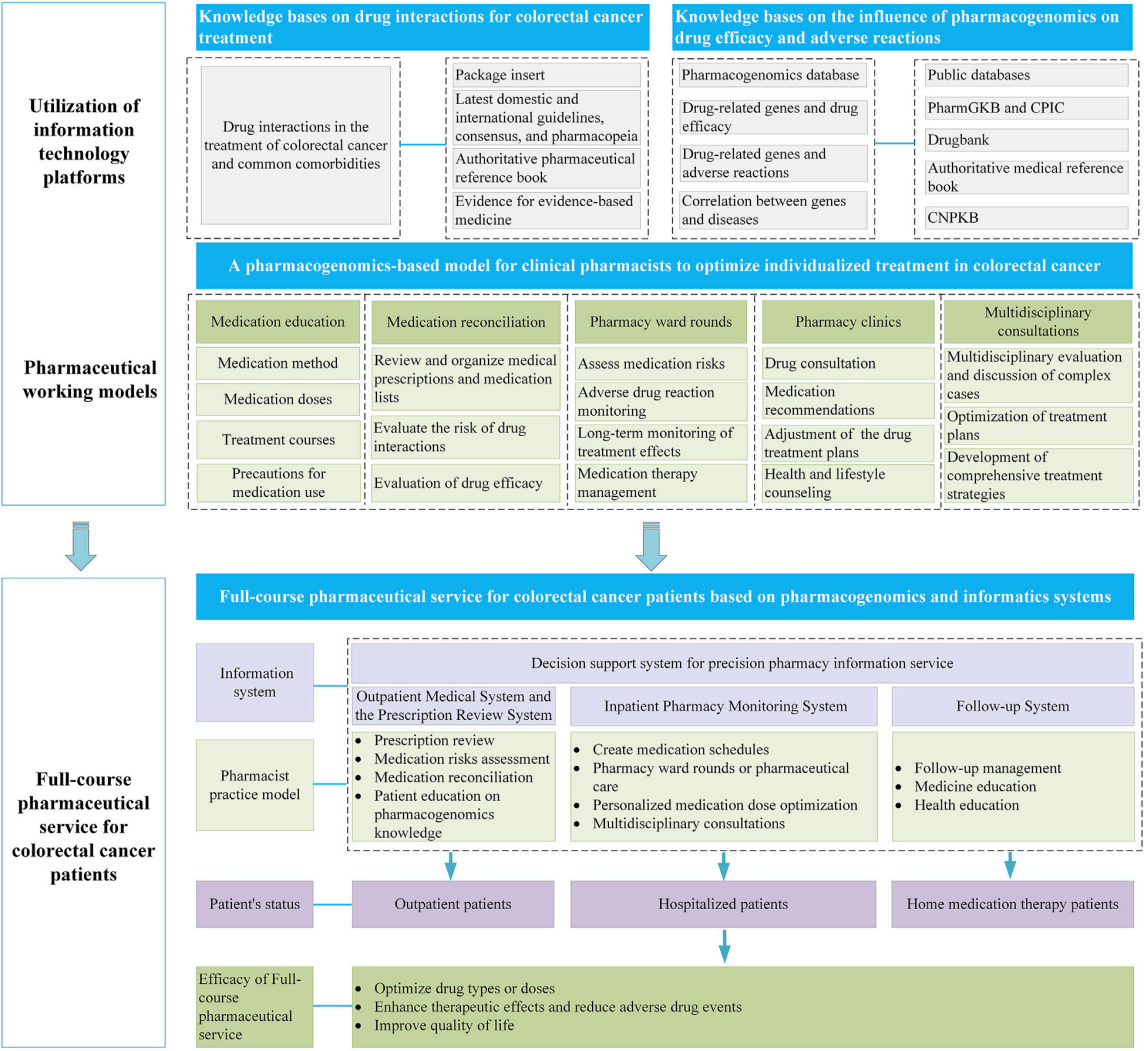
Full-course pharmaceutical service for CRC patients using an information system based on PGx.

The personalized medication systems constructed based on PGx with the utilization of informatic softwares have become increasingly prevalent in clinical settings. Commonly, such systems can issue clinical medication interpretation reports for nearly 100 drugs that are routinely subjected to PGx in clinical practice. Clinical pharmacists can generate such reports with a single click using this kind of system, significantly enhancing work efficiency. Meanwhile, the system incorporates personalized medication models established based on population pharmacokinetics or artificial intelligence techniques. Taking the patient’s clinical information (such as basic patient information, medication records, examination and test information, and surgical records), therapeutic drug monitoring (TDM), and PGx data as input variables, and through matching operations, personalized medication suggestions are provided. Hereinafter, we will conduct a comprehensive analysis from three aspects: outpatient, inpatient, and home-based treatment. With the assistance of information systems, we will explore the role of pharmacists using PGx in different working modes (Figure 2), providing a reference for subsequent personalized pharmaceutical services.

### 3.1 Outpatient therapy

With the continuous introduction of new anti-tumor drug treatment regimens, an increasing number of patients are opting for outpatient therapy. The establishment of pharmacy outpatient clinics has emerged as one of the means by which pharmacists provide effective pharmaceutical services to outpatient patients in recent years (Yang et al., 2022). Oncology pharmacy outpatient clinics have demonstrated certain efficacy in handling ADRs and enhancing patients’ medication compliance (Wang et al., 2022). Thus, in the outpatient setting, pharmacists can make use of the Outpatient Medical System and the Prescription Review System to establish a medication list for CRC patients, review prescriptions to assess potential medication risks, and subsequently adopt medication reconciliation (MR) to assist patients in rational medication use. MR primarily involves creating a comprehensive medication list to record the patient’s all-around medication information. By communicating with the patient and rechecking the medications, it compares whether the patient’s current medication treatment plan is consistent with the doctor’s orders to identify potential medication issues (Powis et al., 2023). The World Health Organization (WHO) recommends that pharmacists should be involved in MR to obtain a more accurate medication list (Kwan et al., 2013; Splawski and Minger, 2016). MR can effectively reduce adverse drug events in tumor patients (Herledan et al., 2020). A randomized controlled study analyzed the influence of MR on the incidence of reconciliation error that reached the patient (RERP) and found that the proportion of RERP occurrence in 147 tumor patients significantly decreased after MR (4% vs. 30%, *p* = 0.0009) (Vega et al., 2016).

Furthermore, pharmacists can recommend to patients whether they need to undertake PGx testing based on their actual medication usage. As the test results of drug genotypes have a latency, some tumor patients may receive drug gene-related medication consultations in the outpatient department. Pharmacists should also be prepared to adjust the drug treatment plans based on the genotype results in the outpatient setting (Cavallari et al., 2016) and simultaneously conduct the popularization and education of PGx knowledge to patients. Petry et al. conducted MR for 465 participants who had recently received or were awaiting pharmacogenetic testing and found that 4.3% of the patients required the re-adjustment of pharmacogenetic recommendations after MR, indicating that MR can further optimize the precise treatment effect of PGx (Petry et al., 2022). Additionally, Cicali et al. summarized the experience of applying PGx in the outpatient department and analyzed the challenges existing in promoting the application of PGx from diverse aspects, such as the optimal sample collection method, the contradiction between waiting for genotype results and the timing of drug administration, and the formulation of relevant clinical medication suggestions, providing a reference for the implementation of PGx in the outpatient department (Cicali et al., 2019).

### 3.2 Inpatient therapy

During the inpatient therapy of CRC patients, pharmacists can employ the Inpatient Pharmacy Monitoring System to draw a medication schedule and reduce the incidence of medication errors in patients through pharmacy ward rounds (evidence level: B, recommendation strength: strongly recommended) (Department of Pharmacy, 2024). Bullock et al. reported that the suitability of patients’ medication significantly improved after pharmacists participated in ward rounds to intervene in patients’ medication (25.4% vs. 36.9%; *p* = 0.004) (Bullock et al., 2019). The research team further evaluated the impact of pharmacists’ participation in patients’ ward rounds on the score of medication risk assessment and found that the proportion of “low and moderate” actionable medication recommendations was higher in the pharmacist intervention group, and patients were more likely to take action based on the recommendations (Bullock et al., 2020). Additionally, pharmacy ward rounds are conducive to adopting more proactive intervention measures and are associated with cost savings (English et al., 2020). For patients with complex conditions, multidisciplinary consultations (MDT) can be conducted during the inpatient therapy. Tumor multidisciplinary consultation is a collaboration among different professionals involved in tumor treatment, providing comprehensive support to tumor patients during diagnosis, treatment, and follow-up, thereby enhancing patients’ treatment compliance and tolerance (Taberna et al., 2020). Tumor patients can receive more precise treatment after MDT (Pillay et al., 2016). Feral et al. compared the differences in ADRs and drug-related problems (DRPs) before and after MDT in patients using anti-tumor drugs for 2 months and found that the number of patients experiencing ADRs (109 cases vs. 41 cases) and DRPs (53 cases vs. 40 cases) decreased after MDT (Feral et al., 2022). When participating in MDT, pharmacists can make personalized medication recommendations based on the patients’ pathophysiological state, laboratory test indicators, examination results, and therapeutic evaluation, regarding the adjustment of chemotherapy drug types or doses. Fan et al. conducted a retrospective study on 812 pharmacist-led consultations and found that the effective rate of consultations increased by 9.3% and the acceptance rate of consultations also increased (6.2%) after pharmacists’ MDT interventions (Fan et al., 2022). During inpatient therapy, pharmacists can play an important role in facilitating precise clinical medication under the guidance of PGx in aspects such as conducting PGx tests, establishing genotyping, constructing user-friendly clinical decision support tools to assist in interpreting genotype test results, and providing clinical decision-making recommendations (Cavallari et al., 2016).

### 3.3 Home-based therapy

With the advent of new drugs, patients in the stable phase can take oral medications at home, and the survival rate of patients keeps increasing. The management of home medication therapy for CRC patients with the participation of pharmacists is becoming increasingly crucial. Home pharmaceutical services mainly revolve around the management of the main therapeutic drugs for CRC and its complications, fulfilling the personalized demands of CRC patients during the medication treatment process, and conducting targeted assessment, analysis, and resolution of potential or existing medication-related issues during the treatment (Pharmacy Today, 2024). Salmani et al. included 469 patients at home undergoing multi-drug therapy and conducted home medication consultations for them (Wang et al., 2019). It was found that after pharmacist intervention, the types of medications taken by patients decreased significantly (*p* < 0.001), and both the level of drug interactions and the DRPs associated with drug interactions decreased significantly (*p* < 0.001), indicating that pharmacist-involved home treatment medication management can enhance the safety of patients’ medication use (Wang et al., 2019). Additionally, the follow-up of discharged patients by pharmacists also plays a significant role in reducing the readmission rate (Phatak et al., 2016b). A prospective randomized controlled study compared the impacts of pharmacist participation in post-discharge follow-up on medication errors and readmission rates and found that the proportion of patients with readmissions or emergency visits after pharmacist intervention was significantly lower (39% vs. 24.8%, *p* = 0.01), and patients on complex medication regimens could benefit significantly from pharmacist intervention (Phatak et al., 2016a). Home pharmaceutical services not only reduce the 90-day readmission rate but also cut down on patients’ medical expenses and hospital admissions (Wiegmann et al., 2020; Zhang et al., 2023). Pharmacists can also offer home medication services by visiting patients, conducting MR and medication education, and providing medication optimization recommendations to ensure the continuity of medication management during the transition from the hospital to the home (Pherson et al., 2014). Patients undergoing home therapy can also use smart apps for medication management, allowing CRC patients to manage their conditions more independently, thereby improving clinical outcomes and reducing hospital visits (Salmani et al., 2022). Therefore, it is suggested that pharmacists analyze the home medication habits, medication efficacy, ADRs, and potential medication risk points of patients based on the patient information collected in the information system. Moreover, in countries such as the United States and Switzerland, pharmacists can also conduct PGx testing in community pharmacies (Haga et al., 2021; Jeiziner et al., 2023). After patients obtain the test results, pharmacists will conduct regular follow-ups to evaluate the drug efficacy and ADRs, answer patients’ doubts regarding drug genetics, and adjust the medications based on changes in other non-genetic factors (Stäuble et al., 2022). Pharmacists can further provide more targeted and precise pharmaceutical services for colorectal cancer by integrating the genetic test results of patients into the informatic softwares. In conclusion, pharmacists play a crucial role in delivering comprehensive and personalized pharmaceutical care to CRC patients through the use of information systems, which effectively support the clinical implementation of PGx.

## 4 Pharmacists participate in the drug management of CRC patients through the information system

Pharmacists’ participation in the pharmaceutical services for CRC patients encompasses various models such as medication education, medication reconciliation, pharmacy rounds, pharmacy clinics, and multidisciplinary consultations, enabling them to provide high-quality pharmaceutical services (Zhen et al., 2019; Zhuang et al., 2021; Feral et al., 2022; Yang et al., 2022; Sun et al., 2024). Currently, under this collaborative model, pharmacists and patients, medical staff, and other professionals in related fields can exchange information through information software and work together to improve the health status and quality of life of patients. The following compares the role of PGx in different treatment schemes for CRC, analyzes the importance of the clinical application of PGx, and discusses the advantages of PGx and information systems in drug management.

Fluorouracil drugs (including 5-FU and its oral prodrug capecitabine), oxaliplatin, and irinotecan (CPT-11) are the first-line chemotherapy drugs for CRC, and their chemotherapy regimens (such as XELOX, FOLFOX, and FOLFIRI) are widely used in clinical practice. FOLFOX and XELOX regimens are similar in that they are both composed of fluorouracil and oxaliplatin. Currently, the evidence level of association between 5-FU, capecitabine, and DPYD gene in the CPIC database and pharmGKB is A and 1A, respectively. The DPYD gene is highly polymorphic and is associated with an increased risk of drug toxicity in CRC patients using fluorouracil (Ruzzo et al., 2017). By comparing the clinical studies of Henricks et al. (2018) and Yamada et al. (2018), it was found that the incidence of grade 3 and above ADR was significantly different in the two CRC study groups with the same treatment regimen containing fluorouracil. In the former study, after the DPYD gene test, the incidence of all grade 3 and above ADR was 23.9%, and the incidences of grade 3 and above gastrointestinal ADR and grade 3 and above hand-foot syndrome were 18.5% and 3.35%, respectively. In the latter study, the incidence of the above ADR in the treatment regimen containing fluorouracil drugs was 64.9%, 9.34%, and 6.20%, respectively, suggesting that drug genes can play a good role in reducing the incidence of serious ADR.

FOLFIRI regimen is the standard chemotherapy regimen for the treatment of CRC and consists of two chemotherapy drugs, irinotecan, and fluorouracil. In addition to fluorouracil, the evidence level of association between irinotecan and the UGT1A1 gene also reached the A and 1A levels in the CPIC database and pharmGKB, respectively. Sanoff et al. (2024) found that the drug gene-guided administration of irinotecan improved PFS in 100 CRC patients treated with FOLFIRI combined with bevacizumab. Compared with the 52 KRAS wild-type subgroup in the study of Izawa et al. (2020) who received the same treatment regimen (FOLFIRI combined with bevacizumab), the median PFS (12.5 months) and median OS (24.5 months) of the former were both longer than those of the latter (median PFS: 5.9 months, median OS: 14.5 months). Similar comparative results were also reflected in the studies of Páez et al. (2018) and Falcone et al. (2007) For CRC patients using the same chemotherapy regimen (FOLFIRI), the treatment based on UGT1A1 gene test results was more effective than that of untested patients (median PFS: 8.6 months vs. 6.9 months, median OS: 26 months vs. 16.7 months), the incidences of grade 3 and above ADR such as neutropenia (17.7% vs. 29%), nausea and vomiting (0% vs. 2%), diarrhea (5.1% vs. 12%) were also significantly reduced. The comparison of these studies highlights the advantages of genetic testing in predicting the efficacy and ADR of chemotherapy drugs for CRC. However, the secondary analysis based on the results of literature studies still has some limitations, and it is impossible to completely calculate the baseline data of the compared patients, which may affect the conclusion. Different sample sizes will also affect the analysis of efficacy and ADR. Therefore, more PGx clinical studies with large sample size and longer follow-up time are needed for verification.

Furthermore, molecular targeted immunotherapy is also one of the main therapies to improve the prognosis of CRC. Before treatment, it is recommended to detect the mutations of KRAS, NRAS, BRAF V600E, and other genes, and the genomic status of Microsatellite (MS) and/or Mismatch repair (MMR) tumors (Bando et al., 2023), to achieve the effect of precise treatment. Among them, it has been confirmed in large clinical studies that cetuximab is more effective in the treatment of CRC when the RAS gene is wild-type (Qin et al., 2018; Heinemann et al., 2020), while bevacizumab is the preferred CRC targeted therapy when RAS and/or RAF genotype mutations occur (Stintzing et al., 2023). PD-1/PD-L1 inhibitors have been shown to be effective in the treatment of most Microsatellite instance-high (MSI-H)/Mismatch repair deficient (dMMR) types of CRC. Immunotherapy methods such as pembrolizumab (Diaz et al., 2022), nivolumab (Overman et al., 2017), and nivolumab combined with ipilimumab (Overman et al., 2018) can effectively improve the prognosis of MSI-H/dMMR CRC patients.

In conclusion, PGx can improve the prognosis of patients and reduce the incidence of ADR, and a PGx-based individualized medication regimen is an effective method to optimize medication management. Pharmacists play a unique role in drug optimization with the help of PGx and information systems. From starting PGx detection to interpreting test results, pharmacists propose treatment suggestions or potential drug use risks to doctors or patients through information systems based on genetic test results. All these measures are effective ways for pharmacists to identify drug differences and make drug adjustments (Zierhut et al., 2017; Haga, 2023).

## 5 Recommendations for personalized medication of CRC based on PGx

The CPIC and PharmGKB are established on the utilization of PGx in clinical medication practice (Caudle et al., 2014; Barbarino et al., 2018). Table 1 presents a comprehensive overview of commonly used chemotherapeutic agents for CRC, including fluorouracil analogs, CPT-11, and oxaliplatin, along with information on drug-associated genes that possess evidence or have been documented in the literature based on current PGx findings. The evidence for fluorouracil analogs and CPT-11 is particularly robust. Tables 2, 3 summarize the recommendations for UGT1A1 that guide the clinical application of CPT-11 as outlined in relevant guidelines and drug labels, retrospectively. Similarly, Table 4 presents the recommendations for DPYD, which inform the clinical utilization of fluorouracil.

**TABLE 1:** Gene information table of chemotherapy drugs for colorectal cancer.

Drug	Gene	Chromosome	Gene sites	CPIC level^(a)^	Pharm GKB level of evidence^(b)^	PGx on FDA level^(c)^	References
fluorouracil	DPYD	1p21.3	rs3918290 (c.1905 + 1G>A)	A	1A	Testing recommended (capecitabine); Actionable PGx (fluorouracil)	CPIC, 2024; GeneCards (2024), PharmGKB (2024)
	MTHFR	1p36.22	rs1801131 (A1298T),rs1801133 (C677T)	—	—	—	Atasilp et al. (2022b)
	NQO1	16q22.1	rs1800566	D	3	—	CPIC, 2024; GeneCards (2024), PharmGKB (2024)
	TYMS	18p11.32	rs45445694,rs11280056, rs2853741,rs183205964	D	3	—	CPIC, 2024; GeneCards (2024), PharmGKB (2024)
	UMPS	3q21.2	rs3772809,rs3772810 rs2291078, rs2279199	D	3	—	CPIC, 2024; GeneCards (2024), PharmGKB (2024)
Irinotecan	UGT1A1	2q37.1	rs4148323, rs10929302	A	1A	Testing recommended	Atasilp et al. (2022a), Hulshof et al. (2022a)
	C8orf34	8q13.2	rs1517114	D	3	—	CPIC, 2024; GeneCards (2024), PharmGKB (2024)
	SEMA3C	7q21.11	rs7779029, rs11979430	D	3	—	CPIC, 2024; GeneCards (2024), PharmGKB (2024)
	SLCO1B1	12p12.1	rs2306283, rs4149056	—	3	—	CPIC, 2024; GeneCards (2024), PharmGKB (2024)
	ABCB1	7q21.12	rs2032582, rs1045642	—	—	—	Li et al. (2020)
Oxaliplatin	GSTM1	1p13.3	GSTM1 non-null, GSTM1 null	D	3	—	CPIC, 2024; GeneCards (2024), PharmGKB (2024)
	GSTP1	11q13.2	rs1695, rs1138272	D	3	—	Li et al. (2020)
	XRCC1	19q13.31	rs25487	—	—	—	Li et al. (2020)

Note: (a): CPIC, Clinical Pharmacogenetics Implementation Consortium; CPIC, assigns seven levels to gene/drug pairs. The levels, in descending order, are A, A/B, B, B/C, C, C/D, and D; (b): Pharm GKB, Pharmacogenetics and Pharmacogenomics Knowledge Base. PharmGKB: assigns six levels to gene/drug pairs. The levels, in descending order, are1A, 1B, 2A, 2B, 3, 4; (c): FDA: Food and Drug Administration. FDA-approved labels of PGx, information are assigned to four levels. The levels, in descending order, are Testing required, Testing recommended, Actionable PGx, and Informative PGx.

Abbreviations: DPYD, dihydropyrimidine dehydrogenase; MTHRF, methylenetetrahydrofolate reductase; NQO1, NAD(P)H quinone dehydrogenase 1; TYMS, thymidylate synthetase; UMPS, uridine monophosphate synthetase; UGT1A1, UDP, Glucuronosyltransferase family one member A1; U8orf34, chromosome eight open reading frame 34; SEMA3C, Semaphorin 3C; SLCO1B1, solute carrier organic anion transporter family member 1B1; ABCB1, ATP, binding cassette subfamily B member 1; GSTM1, glutathione S-transferase mu 1; GSTP1, glutathione s-transferase pi 1; XRCC1, X-Ray repair cross complementing 1.

**TABLE 2:** Medication recommendations for CPT-11 in patients with colorectal cancer based on UGT1A1 guidelines.

Guideline	NM	IM	PM
Phenotype	*1/*1	*1/*28	*28/*28
DPWG^(a)^	The DPWG decided to refrain from a recommendation for *1/*1 (Hulshof et al., 2022c)	No dose reduction when start treatment with irinotecan (Hulshof et al., 2022c)	Start with 70% of the normal dose If the patient tolerates this initial dose, the dose can be increased, guided by the neutrophil count (Hulshof et al., 2022c)
RNPGx/GPCO(b)	Administration of an intensive dose (240 mg/m^2^) is recommended only for *1/*1 patients (Quaranta and Thomas, 2017)	Administration of an intensive dose (240 mg/m^2^) is recommended for *1/*28 patients who have no other risk factors and who benefit from intensive surveillance (Quaranta and Thomas, 2017)	Dose reduction of 25%–30% at the 1st cycle in UGT1A1*28/*28 patients with doses in the 180–230 mg/m^2^ (Quaranta and Thomas, 2017). And *28/*28 patients must not receive high-dose irinotecan (≥240 mg/m^2^) (Quaranta and Thomas, 2017)
AIOM and SIF(c)	—	—	A dose reduction of 30% in *28/*28 patients (RACCOMANDAZIONI PER ANALISI FARMACOGENETICHE, 2024)

Abbreviations: NM, normal metabolizer; IM, intermediate metabolizer; PM, poor metabolizer. (a) DPWG: the Dutch Pharmacogenetics Working Group; (b) RNPGx: the French joint workgroup coming from the National Pharmacogenetic Network (RNPGx) and the Group of Clinical Oncologic Pharmacology (GPCO); (c) AIOM, and SIF: Italian association of medical oncologists (AIOM) and Italian Society of Pharmacology (SIF).

**TABLE 3:** Medication recommendations for CPT-11 in patients with colorectal cancer based on UGT1A1 drug label.

Guideline	PM
Phenotype	*28/*28,*6/*6,*6/*28
FDA	A reduction in the starting dose by at least one level of CAMPTOSAR should be considered for patients known to be homozygous for the UGT1A1*28 allele, the UGT1A1*6 allele or compound heterozygotes (U.S. Food and Drug, Irinotecan hydrochloride, 2024) The recommended starting dose of ONIVYDE in patients known to be homozygous for the UGT1A1*28 allele is 50 mg/m^2^ administered by intravenous infusion. Increase the dose of ONIVYDE to 70 mg/m^2^ as tolerated in subsequent cycles (U.S. Food and Drug, Onivyde, 2024)
EMA(a)	A reduced starting dose of ONIVYDE (liposomal irinotecan) of 50 mg/m2 should be considered for patients with the UGT1A1*28/*28 genotype A dose increase of ONIVYDE to 70 mg/m2 should be considered if tolerated in subsequent cycles (European Medicines Agency, 2024)
HCSC(b)	A reduced irinotecan starting dose should be considered for patients known to be homozygous for UGT1A1*28 allele (HCSC, 2024)
Swissmedic(c)	In patients who are homozygous carriers of UGT1A1*28, the recommended initial dose of ONIVYDE (liposomal irinotecan) is 60 mg/m^2^. An increase in dose from ONIVYDE to 80 mg/m^2^ may be considered if tolerated for subsequent cycles (Swissmedic, 2020)

Abbreviations:(a) EMA, European Medicines Agency; (b) HCSC, Health Canada (Santé Canada); (c) Swissmedic: Swiss Agency for Therapeutic Products.

**TABLE 4:** Medication recommendations for fluoropyrimidines^(a)^ in patients with colorectal cancer based on DPYD.

Guideline	NM	IM	PM
Phenotype	*1/*1	*1/*2A, *1/*13	*2A/*2A, *13/*13, *2A/*13
Activity score	2	1, 1.5	0, 0.5
DPWG	Standard dose (Lunenburg et al., 2020)	Start with 50% of the standard dose of 5-fluorouracil or capecitabine (Lunenburg et al., 2020)	Subjects with a gene activity score of 0 are recommended to avoid both systemic and cutaneous 5-fluorouracil or capecitabine (Lunenburg et al., 2020)
RNPGx	Standard dose	Dose should be reduced 50% for the first cycle ((RNPGx))	Fluoropyrimidines are contraindicated due to the risk of fatal toxicity with the standard dose ((RNPGx))
CPIC	Based on genotype, there is no indication to change dose or therapy. Use label-recommended dose and administration (Amstutz et al., 2018)	Activity score 1: Reduce dose by 50% Activity score 1.5: Reduce dose by 25%–50% (Amstutz et al., 2018)	Activity score 0.5: Avoid use of 5fluorouracil or 5-fluorouracil prodrug-based regimens Activity score 0: Avoid use of 5-fluorouracil or 5-fluorouracil prodrug-based regimens (Amstutz et al., 2018)
SEFF/SEOM^(b)^	—	Reduce starting dose by 50% followed by titration of dose based on toxicity or pharmacokinetics	Contraindicated treatment with fluoropyrimidines; look for alternative agents
FDA	—	Insufficient data are available to recommend a dose in intermediate metabolizers	No dose has proven safe in poor metabolizers

Abbreviations: DPWG, dutch pharmacogenetics working group; RNPGx, French National Network of Pharmacogenetics; CPIC, clinical pharmacogenetics implementation consortium; FDA, U.S., Food and Drug Administration.(a) 5-fluorouracil or capecitabine; (b) SEFF/SEOM: Spanish Pharmacogenetics and Pharmacogenomics Society (SEFF) and Spanish Society of Medical Oncology (SEOM).

Nevertheless, there is currently no consensus regarding the necessity of genotyping in patients receiving low-dose CPT-11. The RNPGx/GPCO do not advocate for UGT1A1 genotyping in patients administered low-dose (<180 mg/m^2^) CPT-11 prior to treatment, citing similarities in hematologic and gastrointestinal toxicity at these doses regardless of CPT-11 genotype. Conversely, the DPWG asserts that even low-dose CPT-11 carries an increased risk of grade 3-4 neutropenia (Hu et al., 2010; Liu et al., 2014), thereby recommending genotyping for all patients undergoing CPT-11 treatment. Further multi-center studies encompassing diverse dosing regimens are needed to substantiate this issue.

According to the guideline of Chinese Society of Clinical Oncology (CSCO) for colorectal cancer in 2024, it is recommended to decrease the dose of CPT-11 for patients carrying the homozygous or double heterozygous for UGT1A1*28 or UGT1A1*6. Specifically, for patients with wild-type UGT1A1, the recommended CPT-11 dose when utilizing the mXELIRI regimen is 200 mg/m2. Conversely, for those who are homozygous for UGT1A1*28 or UGT1A1*6 or double heterozygous for both UGT1A1*28 and UGT1A1*6, the recommended CPT-11 dose is reduced to 150 mg/m^2^. The National Comprehensive Cancer Network (NCCN) Clinical Practice Guidelines in Oncology: Colon Cancer (Version 5.2024) similarly states that the dose of CPT-11 should be individualized based on the UGT1A1 genotype and that the initial dose of the drug should be reduced for patients identified as carriers of UGT1A1*28 purists. Additionally, distinct recommended dosing regimens have been established in the guidelines for targeted drugs and immune checkpoint inhibitors (ICIs) with varying drug targets in patients with CRC, as outlined in Table 5. To sum up, although the current evidence levels for guiding the clinical dose adjustment of CPT-11 and fluoropyrimidine drugs based on PGx are relatively high, there are slight differences in the suggestions of guidelines in various countries. Additionally, at present, the mutation frequency of the relevant high-evidence-level drug genes (such as DPYD) of fluoropyrimidine drugs is relatively low in the East Asian population. In practical clinical applications, it is impossible to directly refer to the opinions of the guidelines. Instead, it is necessary to conduct a comparative analysis in combination with the clinical research results of a large sample size of the Chinese population. Therefore, establishing a personalized medication regimen suitable for Chinese patients based on the existing PGx guidelines is one of the crucial steps in optimizing the individual treatment of CRC patients.

**TABLE 5:** Medication recommendations for targeted agents and ICIs in patients with colorectal cancer.

Guideline/Genotypes	2024CSCO	NCCN 2024.V5 colon cancer/NCCN 2024.V4 Rectal Cancer	ESMO
dMMR/MSI-H	Pembrolizumab Pucotenlimab Nivolumab Nivolumab + Ipilimumab Envafolimab Serplulimab Tislelizumab	Pembrolizumab Nivolumab Nivolumab+Ipilimumab Dostarlimab-gxly	Pembrolizumab Nivolumab + Ipilimumab
POLE/POLD1 mutation		Pembrolizumab Nivolumab Nivolumab+Ipilimumab Dostarlimab-gxly	
HER2 amplification	Trastuzumab + Pertuzumab Trastuzumab + Lapatinib	Famtrastuzumab deruxtecan-nxki	Trastuzumab-lapatinib Trastuzumab-pertuzumab Trastuzumab- trastuzumab Deruxtecan
HER2 amplification and RAS、BRAF wild-type	—	Trastuzumab + Pertuzumab Trastuzumab + Lapatinib Trastuzumab + Tucatinib	
RAS mutation	—	Cetuximab/Panitumumab+Sotorasib/Adagrasib (KRAS G12C mutation)	Avoid using cetuximab and anitumumab
RAS wild-type/BARF V600E mutation	Vemurafenib + Irinotecan + Cetuximab	—	
KRAS/NRAS/BRAF wild-type	—	Cetuximab or Panitumumab Cetuximab Panitumumab	
BRAF V600E mutation positive	—	Encorafenib + Cetuximab Encorafenib + Panitumumab	Encorafenib-cetuximab
NTRK fusions positive	—	Larotrectinib Entrectinib Repotrectinib	Larotrectinib Entrectinib
RET fusions positive	—	Selpercatinib	—
ALK or ROS1 fusions positive	—	—	Entrectinib

ESMO: The European Society for Medical Oncology.

## 6 Conclusion

Genetic factors are a significant contributor to inter-individual variability in drug response, with an estimated influence of 20%–30% on the overall observed variation. The remaining 70%–80% or more of the observed variation is attributed to non-genetic factors such as the patient’s pathophysiological status, the presence, and stage of comorbid conditions, drug-drug interactions, and individual factors (gender, age, weight, nutritional status, lifestyle habits). The multiplicity of contributing factors also gives rise to discrepancies between the findings of pharmacogenetic research and the recommendations set forth in clinical guidelines in actual clinical practice. In the context of implementing individualized precision medicine, the results of pharmacogenetic studies are insufficient to comprehensively and objectively reflect the differences in drug efficacy and/or ADRs observed among different patients, which may lead to “black box treatment suggestions”. Therefore, providing relatively effective personalized medication suggestions still requires integrating multi-omics genetic factors based on clinical data and utilizing information technology software to integrate a plethora of information. This paper presents the rational medication information established based on PGx evidence and a multi-omics data analysis collaboration platform combined with clinical data, along with suggestions for pharmacists’ participation in clinical medication decision-making. Taking CRC as an example, we analyze the practical processes of pharmacists in different pharmacy working models by combining information systems and PGx, and their impacts on patient efficacy and ADRs. Finally, based on the existing guideline results, more comprehensive and personalized drug treatment suggestions are formulated for different populations. In conclusion, the integration of information systems is conducive to overcoming some obstacles in the clinical implementation of PGx in the future and facilitating personalized healthcare.
